# The meaningfulness of searching for minimal exposure duration to understand visual perception

**DOI:** 10.1038/s41467-026-75041-5

**Published:** 2026-07-09

**Authors:** Pascal Mamassian, Mark Wexler

**Affiliations:** 1 Laboratoire des Systèmes Perceptifs, Département d’études Cognitives, École Normale Supérieure, PSL University, CNRS, Paris, France; 2 Integrative Neuroscience and Cognition Center, Université Paris Cité, CNRS, Paris, France

**Keywords:** Human behaviour, Pattern vision

**arising from** R. C. Lanfranco et al*. Nature Communications* 10.1038/s41467-024-52778-5 (2024)

Two centuries ago, Joseph Plateau remarked that when a piece of burning coal moves quickly in the dark, we see a continuous luminous streak, thus proving that the sensations produced by light have a certain duration^1^. The term “visual persistence” was later coined to describe the phenomenon that the visual perception of an object lingers for some time after its physical disappearance. This perceived duration varies with some stimulus properties, most notably its luminance, but it cannot be below a critical value known as the flicker fusion threshold. This threshold in humans is estimated to be around one sixtieth of a second^2^, so that two flashes separated by less than about 15 ms cannot be distinguished. In light of these well-established phenomena, when Lanfranco and collaborators^3^ report some behavioural effects for durations as short of 0.4 ms (see their Experiment 3), their results may not be related to duration per se.

Visual persistence is usually explained by the fact that the visual system has a finite integration time. While the exact time constants of this temporal integration are not completely settled, there is little doubt that the thresholds reported by Lanfranco and collaborators^3^ fall well within this temporal window. A manifestation of temporal integration is Bloch’s law^4,5^: the effective luminosity or contrast of brief stimuli grows approximately linearly with their duration. While there has been some question about the applicability of Bloch’s law to complex stimuli^6^, as pointed out by the authors on p. 54 of their Supplementary materials, we have directly verified that it holds for Lanfranco et al.’s exact facial stimuli (Fig. 1). This is in agreement with the subjective impression of these stimuli: at the briefest durations they appear to have extremely low contrast and no internal features; as the duration increases, apparent contrast increases and facial features emerge; but for durations below 30–40 ms, all stimuli appear to have the same duration, in agreement with Bloch’s law. Thus, the simplest explanation of the results of Lanfranco et al. is that, by manipulating stimulus duration, they are effectively manipulating stimulus contrast instead. Indeed, the visual system that features a low-pass filter, cannot distinguish between different brief durations, other than through integrated contrast. At very low contrasts resulting from brief stimulus durations, the visual system cannot distinguish between normal and scrambled faces; but an upright face may have a lower contrast threshold than an upside-down face (their Experiment 1).

**Fig. 1:**
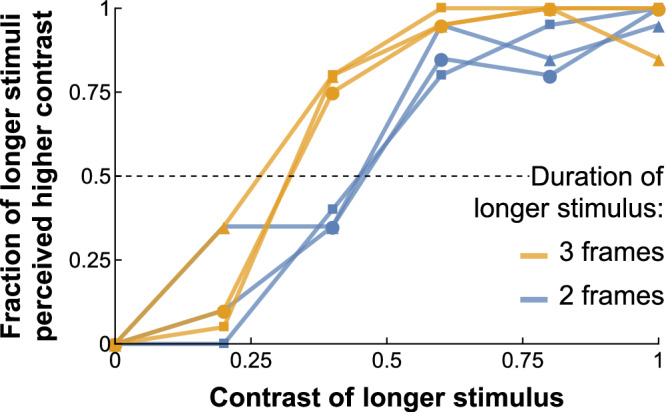
Bloch’s law for face stimuli used by Lanfranco et al. Face stimuli were presented on a calibrated 360 Hz OLED monitor at full contrast for a single frame (2.8 ms). In a forced-choice procedure, three participants (different symbols) compared the contrast of this stimulus to that of a longer stimulus with reduced contrast. The longer stimuli lasted either 2 frames (5.6 ms; blue curves) or 3 frames (8.3 ms; orange curves). The points of subjective contrast equality were on average 45% for 2 frames and 32% for 3 frames, close to the values of 50% and 33% predicted by Bloch’s law.

Another important aspect to keep in mind when considering brief stimuli is how long they are accessible to the observer. Because of visual persistence, briefly presented stimuli create a short-lived visual representation of visual information called iconic memory^7^. Access to the information contained in iconic memory long after the stimulus has disappeared has been clearly demonstrated, in particular using the partial report paradigm^8^. Iconic memory compensates for brief stimuli by lasting longer, so that the total duration of stimulus availability is roughly independent of the duration of brief stimuli^9^. Therefore, while the manipulation of stimulus duration is important to understand the mechanisms of perception, it is also important to consider how the stimulus is processed in the early stages of visual processing^10^. Ultimately, the aim of determining an absolute minimal exposure duration to reveal important properties of the visual system may be of limited value.

## Methods

The present study was approved by the ethics committee of CPP Ouest IV-Nantes (protocol 52/20_3). All participants provided informed consent before running the experiment. Three observers participated in this study, one author and two naive participants, all with normal or corrected to normal visual acuity (1 female, median age 33). No sex- or gender-based analyses were performed because there were no hypotheses concerning sex or gender. Face stimuli were presented on a calibrated 360 Hz OLED monitor at full contrast for a single frame (2.8 ms; 180 cd/m^2^) and then replaced by uniform grey (220 cd/m^2^), duplicating the reported luminance values and other stimulus parameters of Lanfranco and collaborators^3^. In a forced-choice procedure, participants compared the contrast of this stimulus to that of a longer stimulus with reduced contrast. The longer stimuli lasted either 2 frames (5.6 ms) or 3 frames (8.3 ms). Each condition was repeated 20 times for each participant. We checked that each frame in these conditions had the same luminance as the single frame condition on the monitor (because our photometer had a time constant of several hundreds of milliseconds, we measured the mean luminance of alternating single, pairs, or triplets of face and grey frames, and found nearly identical values of 203, 203, and 202 cd/m^2^, respectively). No data were excluded from the analyses. From the psychometric functions plotting the fraction of times the longer stimulus was perceived with higher contrast as a function of the contrast of the longer stimulus, we extracted the point of subjective contrast equality as the contrast value needed to reach 50% on the y-axis.

### Reporting summary

Further information on research design is available in the Nature Portfolio Reporting Summary linked to this article.

## Supplementary information

Reporting Summary

## Data Availability

The data generated in this study have been deposited in the OSF database 10.17605/OSF.IO/2SBG9.

## Code availability

The software to analyze the data in this study has been deposited in the OSF database 10.17605/OSF.IO/2SBG9. The code runs in Mathematica version 12.3.
