# Supplementary material for: The meaningfulness of searching for minimal exposure duration to understand visual perception
Source: Nat Commun. 2026 Jul 9;17:5976. doi: 10.1038/s41467-026-75041-5 (PMC13350935; doi:10.1038/s41467-026-75041-5)
Supplement: Supplementary file 1 — Reporting Summary [file 41467_2026_75041_MOESM1_ESM.pdf]

nature portfolio

Corresponding author(s): Pascal Mamassain
Last updated by author(s): Mar 16, 2026

Reporting Summary

Nature Portfolio wishes to improve the reproducibility of the work that we publish. This form provides structure for consistency and transparency in reporting. For further information on Nature Portfolio policies, see our Editorial Policies and the Editorial Policy Checklist.

Statistics

For all statistical analyses, confirm that the following items are present in the figure legend, table legend, main text, or Methods section.

n/a	Confirmed	
☐	☒	The exact sample size (*n*) for each experimental group/condition, given as a discrete number and unit of measurement
☐	☒	A statement on whether measurements were taken from distinct samples or whether the same sample was measured repeatedly
☒	☐	The statistical test(s) used AND whether they are one- or two-sided<br>*Only common tests should be described solely by name; describe more complex techniques in the Methods section.*
☐	☒	A description of all covariates tested
☒	☐	A description of any assumptions or corrections, such as tests of normality and adjustment for multiple comparisons
☒	☐	A full description of the statistical parameters including central tendency (e.g. means) or other basic estimates (e.g. regression coefficient) AND variation (e.g. standard deviation) or associated estimates of uncertainty (e.g. confidence intervals)
☒	☐	For null hypothesis testing, the test statistic (e.g. *F*, *t*, *r*) with confidence intervals, effect sizes, degrees of freedom and *P* value noted<br>*Give P values as exact values whenever suitable.*
☒	☐	For Bayesian analysis, information on the choice of priors and Markov chain Monte Carlo settings
☒	☐	For hierarchical and complex designs, identification of the appropriate level for tests and full reporting of outcomes
☒	☐	Estimates of effect sizes (e.g. Cohen's *d*, Pearson's *r*), indicating how they were calculated

Our web collection on statistics for biologists contains articles on many of the points above.

Software and code

Policy information about availability of computer code

Data collection	Custom software was used to display the images on our display. Any application that will display an image on a computer monitor while controlling its presentation duration will do.
Data analysis	Very basic data analyses were performed using Mathematica 12.3: data was fitted with logistic functions.

For manuscripts utilizing custom algorithms or software that are central to the research but not yet described in published literature, software must be made available to editors and reviewers. We strongly encourage code deposition in a community repository (e.g. GitHub). See the Nature Portfolio guidelines for submitting code & software for further information.

Data

Policy information about availability of data

All manuscripts must include a data availability statement. This statement should provide the following information, where applicable:

- Accession codes, unique identifiers, or web links for publicly available datasets
- A description of any restrictions on data availability
- For clinical datasets or third party data, please ensure that the statement adheres to our policy

Mean data by participant and condition are displayed in the figure of our manuscript. Full raw data is available in the open repository https://osf.io/2sbg9/

Research involving human participants, their data, or biological material

Policy information about studies with human participants or human data. See also policy information about sex, gender (identity/presentation), and sexual orientation and race, ethnicity and racism.

Reporting on sex and gender	There was no consideration of either sex or gender of participants. No sex- or gender-based analyses were performed because there were no hypotheses concerning sex or gender.
Reporting on race, ethnicity, or other socially relevant groupings	No socially constructed or socially relevant categorization variables were used.
Population characteristics	See above
Recruitment	One of the participants was a co-author; the other two were colleagues who were available on the day that the study was performed. These latter two participants were naive as to the hypotheses of the study.
Ethics oversight	The study was approved by the CPP (Comité de Protection des Personnes) Ouest IV-Nantes (protocol 52/20_3).

Note that full information on the approval of the study protocol must also be provided in the manuscript.

Field-specific reporting

Please select the one below that is the best fit for your research. If you are not sure, read the appropriate sections before making your selection.

☐ Life sciences ☒ Behavioural & social sciences ☐ Ecological, evolutionary & environmental sciences

For a reference copy of the document with all sections, see nature.com/documents/nr-reporting-summary-flat.pdf

Behavioural & social sciences study design

All studies must disclose on these points even when the disclosure is negative.

Study description	In this study participants viewed pairs of brief visual stimuli that differed in their durations and contrasts, and reported which of the two seemed to have higher contrast.
Research sample	There were three participants. One of the participants was a co-author; the other two were colleagues who were available on the day that the study was performed. Two were men and one was a woman. Ages were 28-57, median 33. The sample is in no way representative of the human population, but given the facts that all three participants had very similar results, that the experiment was very simple and constituted a quick counter-demonstration of a statement in the target article, and the general research practices in the field of psychophysics, we feel that the sample is adequate.
Sampling strategy	As stated above, the three participants were one of the co-authors and two readily available colleagues. The two colleagues were chosen randomly while walking down the laboratory hallway. This sampling procedure is commonly employed in our research field. A sample size of 3 is appropriate when little between-participant variation is expected (as indeed was the case). All the participants ran all the experimental conditions.
Data collection	Data was quantitative. On each trial of a forced-choice procedure, participants compared the contrast of a standard stimulus to that of a longer stimulus with reduced contrast. Data was collected using a computer mouse. After several initial practice trials (not analyzed), no one other than the participant was present in the experimental room. The researcher (who was also one of the 3 participants) was not blind to the experimental conditions and hypotheses.
Timing	Data was collected over two days (1 participant on the first day, 2 on the second). Each participant performed two blocks of 100 trials per block. Each block lasted about 4-5 minutes, with 1-2 minutes break between the two blocks.
Data exclusions	No data were excluded
Non-participation	No participants dropped out
Randomization	There were experimental groups (this was a within-participant design)

Reporting for specific materials, systems and methods

We require information from authors about some types of materials, experimental systems and methods used in many studies. Here, indicate whether each material, system or method listed is relevant to your study. If you are not sure if a list item applies to your research, read the appropriate section before selecting a response.

Materials & experimental systems

n/a	Involved in the study
☒	☐ Antibodies
☒	☐ Eukaryotic cell lines
☒	☐ Palaeontology and archaeology
☒	☐ Animals and other organisms
☒	☐ Clinical data
☒	☐ Dual use research of concern
☒	☐ Plants

Methods

n/a	Involved in the study
☒	☐ ChIP-seq
☒	☐ Flow cytometry
☒	☐ MRI-based neuroimaging

Plants

Seed stocks	*Report on the source of all seed stocks or other plant material used. If applicable, state the seed stock centre and catalogue number. If plant specimens were collected from the field, describe the collection location, date and sampling procedures.*
Novel plant genotypes	*Describe the methods by which all novel plant genotypes were produced. This includes those generated by transgenic approaches, gene editing, chemical/radiation-based mutagenesis and hybridization. For transgenic lines, describe the transformation method, the number of independent lines analyzed and the generation upon which experiments were performed. For gene-edited lines, describe the editor used, the endogenous sequence targeted for editing, the targeting guide RNA sequence (if applicable) and how the editor was applied.*
Authentication	*Describe any authentication procedures for each seed stock used or novel genotype generated. Describe any experiments used to assess the effect of a mutation and, where applicable, how potential secondary effects (e.g. second site T-DNA insertions, mosiacism, off-target gene editing) were examined.*
